# Investigation on the plasmon Talbot effect of finite-sized periodic arrays of metallic nanoapertures

**DOI:** 10.1038/srep45573

**Published:** 2017-03-28

**Authors:** Wenli Li, Haoyong Li, Bo Gao, Yiting Yu

**Affiliations:** 1 Key Laboratory of Micro/Nano Systems for Aerospace (Ministry of Education), Northwestern Polytechnical University, Xi’an 710072, China; 2 Key Laboratory of Micro- and Nano-Electro-Mechanical Systems of Shaanxi Province, Northwestern Polytechnical University, Xi’an 710072, China

## Abstract

We present an in-depth and systematical investigation on the plasmon Talbot effect of finite-sized two-dimensional (2D) periodic metallic nanoaperture arrays. The nanoaperture shapes, fill factor, lattice distribution, array size, film thickness, material property and polarization state of the incident light are considered, and the inherent influencing rules are summarized via the three-dimensional (3D) finite-difference time-domain (FDTD) numerical simulations. The nanoaperture shapes, fill factor or array size seems to express a tiny influence on Talbot effect, which shows a good agreement with our previously reported experimental results. Besides, square lattice brings out a much more uniform Talbot pattern than the triangular distribution, and the smaller array period should be taken to estimate the Talbot distance when it comes to a rectangular distribution. Furthermore, the thickness of Au film is suggested to within the range of 50~100 nm, which gives a broadest Talbot contour. It is also found out that the elliptical shape of hotspots is closely related to the linearly polarization state of the light source, showing an asymmetric electromagnetic field. The research contributes to a better understanding of the optical transmission features through periodic metallic nanoaperture arrays, which provides opportunities for the potential applications such as nanofabrication, optoelectronics, and imaging.

Talbot effect, a famous phenomenon existing in the classical optics first discovered by H. F. Talbot in 1836^1^, describes that a periodic structure illuminated by a monochromatic plane wave can produce the optical fields at multiple typical distances replicating exactly the device pattern. The elemental distance was named as the Talbot distance (*τ*). So far, the Talbot effect has been widely utilized and surveyed in the areas of acoustics^2^, scanning electron microscope imaging^3^, X-ray^4^, Bose-Einstein condensers^5^, optical trapping^6^, massless Dirac fermions^7^,^8^, and nonlinear optics^9^,^10^. More recently, the plasmon Talbot effect of periodic metallic nanostructures was explored both theoretically^11^,^12^,^13^ and experimentally^14^,^15^,^16^, showing the potential applications in the fields of high-resolution imaging^17^ and micro-/nanofabrication^18^,^19^. Different from the traditional plasmon effects, such as the localized surface plasmon resonance (LSPR)^20^ and surface-enhanced Raman scattering (SERS)^21^ which are obtained in the near field, the plasmon Talbot effect can even be observed in the far field, forming the exotic Talbot carpet filled with rich subwavelength hotspots^22^.

Recently, Odom *et al*.^23^ studied the finite-sized two-dimensional (2D) periodic metallic arrays called as the “patches”, but they didn’t observe the Talbot effect and attributed it to the finite size of the arrays. Later, Chowdhury *et al*.^24^ experimentally demonstrated the plasmon Talbot effect of the finite-sized 2D periodic arrays of nanoholes, and drawn the conclusion that the incident wavelength must be smaller than the array period to generate the Talbot effect. They found that a larger ratio of the period to wavelength makes the Talbot effect more pronounced. The same viewpoint was verified in our recent report^25^, and furthermore, the influences of structural parameters, including the fill factor, array size, lattice distribution, and nanoaperture shapes on the focusing and Talbot effects of the finite-sized 2D periodic metallic nanoaperture arrays were also experimentally investigated^26^, from which we know that a larger array size shows no obvious influence on the average size of hotspots in the Talbot carpets, and the different nanoaperture shapes exhibit no big difference in the Talbot fields.

Note that there exists some uncertainty about the preliminary experimental results we acquired, taking the manufacturing and testing errors into account. Indeed, to fabricate the micro-optic or nanophotonic devices with a high precision is still a worldwide technological challenge up to date, which pours a large impact on the final experimental results and explains quite well the deviation existing between the theory and practice. In order to thoroughly study for a better understanding of the influences of structural parameters on the Talbot effect and also the other possible factors that may function on the Talbot effect, the rigorous three-dimensional (3D) finite-difference time-domain (FDTD) numerical simulation method is employed in this research, and the nanoaperture shapes, fill factor, lattice distribution, array size, film thickness, material property and polarization state of the incident light are all investigated. To some extent, this research will provide an important technological reference for designing the finite-sized 2D periodic arrays of planar metallic nanostructures for such applications as nanolithography and high-resolution microscopy imaging.

## Modeling and Design

**Figure 1:**
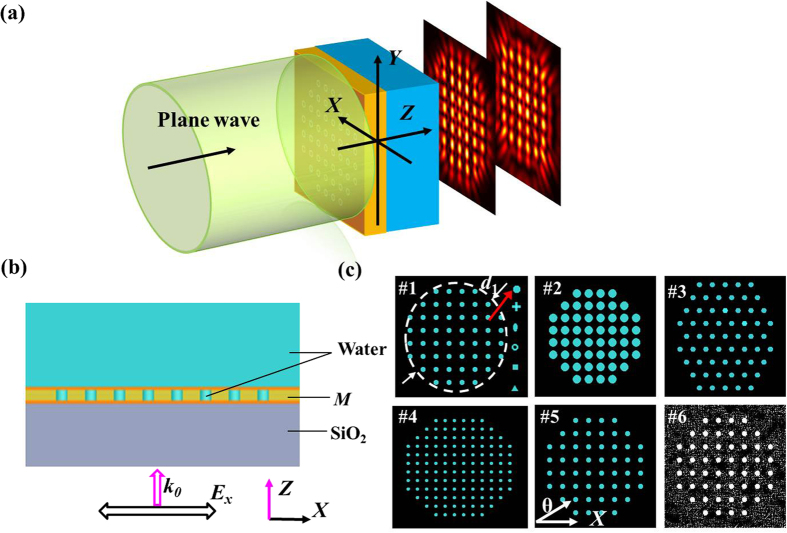
(**a**) Schematic illustration of Talbot effect when the monochromatic plane wave passes through the finite-sized 2D periodic arrays of metallic nanospertures. (**b**) Cross-sectional schematic of the analytical model employed in FDTD simulations. (**c**) Top view of the surveyed samples, #1–#6 representing for different nanoaperture shapes, fill factor, lattice distribution, array size, polarization state, and film thickness or material property, respectively.

When the finite-sized 2D periodic arrays are illuminated by a monochromatic plane wave, a light pattern with complex subwavelength hotspots imitating the exact structure of irradiated device can be intuitionally seen at some Talbot distances, as shown in Fig. 1(a). Figure 1(b) gives the cross-sectional view of the analytical model for the designed planar metallic nanoaperture arrays, and a full-wave 3D electromagnetic simulation was performed using the FDTD Solutions package. The whole structure was normally illuminated by a linearly polarized plane wave of 525 nm wavelength with the wave vector *k*_*0*_ in the *Z* direction and having the electric-field component *E*_*x*_. In this research, the operating wavelength (525 nm) in the air is slightly larger than the array period (500 nm), thus all the high-order interfered diffractions are suppressed when the devices are illuminated, making the Talbot effect invisible. However, water was taken as the output medium to make the effective wavelength less than the array period, and the Talbot effect could take place. This case was specially designed. The studied metallic nanoaperture arrays were of subwavelength periods and etched in the 200 nm-thick gold film which was deposited on the glass wafer of 420 μm in thickness. The samples can be prepared exactly the same as our previous experiments reported in ref. 26. During the process of simulations, perfectly matched layer was applied to all the outermost borders and the mesh size was all set to 10 nm in the three axial directions. The values of dielectric constants for the dispersive metallic material *M* were taken from the Chemical Rubber Company (CRC) datasheet^27^. Figure 1(c) shows the top view of the analytical models for the planar metallic microarrays, among which each sample represents a kind of influencing parameters, such as nanoaperture shapes, fill factor, lattice distribution, array size, polarization state of the incident light, and film thickness or material property.

In Fig. 1(c), sample #1 denotes the six different nanoaperture shapes owning the same opening area, including nanoholes, nanocrosses, nanoellipses, nanorings, nanosquares, and nanotriangles. The arrayed structures can be approximately treated as circles, e.g. with the diameter *d*_1_ for the sample #1, and the array periods in both axes are 500 nm for all the cases. Initially, square lattice distribution is assumed in the following discussion. Besides, sample #2 clearly shows the varying fill factor, by increasing the opening size of nanoapertures. Sample #3 represents the triangular lattice distribution of nanoapertures while sample #4 stands for the change of the total array size of finite-sized 2D periodic arrays. Furthermore, sample #5 constructs the polarization angle *θ* (the angle between the polarization direction and *X* axis) of the incident light. Finally, we investigate the film thickness and material property, illustrated as the sample #6.

## Results and Discussions

In this work, a systematical investigation of various factors, like the nanoaperture shapes, fill factor, lattice distribution, array size, film thickness, material property, and polarization state of the incident light, on the Talbot effect of finite-sized 2D periodic arrays of metallic nanoapertures is performed. To make it clearer, only one factor is considered for the specific simulations, while keeping the others unchanged.

The self-imaging *revivals* produced by the Talbot effect will repeat at multiples of a characteristic distance, the so-called Talbot distance *τ*. When the array period is between *λ*_*e*_ and 2*λ*_*e*_, where *λ*_*e*_ denotes the effective working wavelength calculated by *λ*/*n (n* being the refractive index of the medium), the paraxial approximation is satisfied, and the theoretical Talbot distance *τ* can be estimated by ref. 28


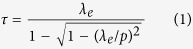


where *p* is the array period and when it approaches infinity, the Talbot distance will equal to 2*p*^2^/*λ*_*e*_, which becomes the classical formula. Accordingly, the Talbot distance calculated is about 1.02 μm, as the array period is of 500 nm for all the samples.

### Nanoaperture shapes

**Figure 2:**
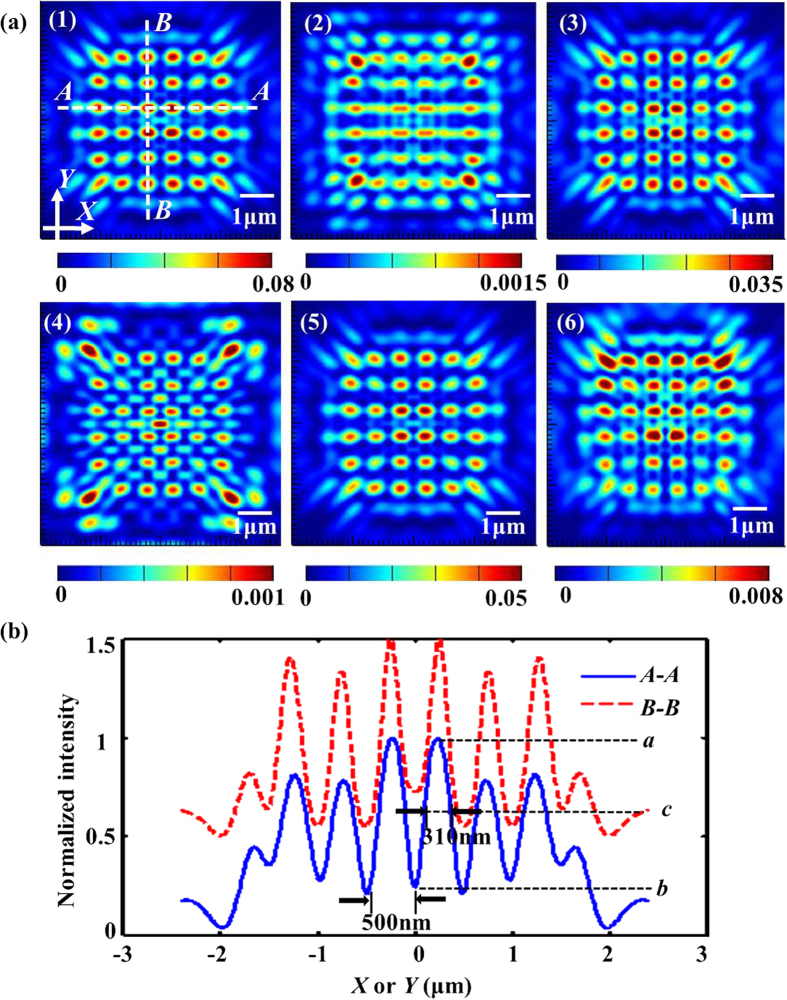
(**a**) The simulated electric-field intensity of various nanoaperture shapes at the first Talbot planes, (1–6) representing for the samples composed of nanoholes, nanocrosses, nanoellipses, nanorings, nanosquares, and nanotriangles, respectively. (**b**) The derived intensity profiles along the *A-A* and *B-B* directions to calculate the average size of hotspots. For clarity, the intensity profile for *B*-*B* is shifted by 0.5 μm in *Y* axis.

In order to study the influence of different nanoaperture shapes on the Talbot effect of finite-sized periodic arrays of metallic nanoapertures, six distinct kinds of nanoaperture shapes drilled in the gold film and arranged in a square lattice were taken into account, denoted in Fig. 1(c) as the sample #1. For simplicity, the first self-imaging positions were investigated, and the derived contours of the optical fields parallel to *X-Y* plane are shown in Fig. 2(a), from which few differences of the hotspots at the first Talbot planes can be seen for the six kinds of nanoaperture shapes. Then, the sizes of hotspots in both axes are further analyzed. Taking the sample (1) for instance, along the dashed lines *A*-*A* and *B*-*B* as shown in Fig. 2(a), the intensity profiles are derived (see Fig. 2(b)), from which the sizes of hotspots can be calculated according to the following method. Firstly, two lines are drawn, *a* and *b*, representing the peak intensity and the average intensity of the two valleys adjacent to a hotspot. After that, a third line *c* is drawn of the middle intensity between the preceding two lines, and the width cut out by the line *c* on the intensity contour gives the actual size of the hotspot. In this paper, the average size of the hotspots at a specific position is concerned, and more than five hotspots are calculated to go for an average value. The simulated results are listed in Table 1, number (*X* or *Y*) implies the average size of hotspots in the *X* or *Y* axis. The periodic repetitions of the optical fields imitating the device patterns can propagate to the far field. However, due to the metallic absorption and propagation loss in the outside medium, the Talbot *revivals* become very weak after several repetitions. The simulated Talbot distance can be calculated according to the several intervals along the optical axis which is also given in Table 1.

Table 1 tells us that the nanoaperture shapes show a negligible influence on the average size of hotspots for both axes. The reason why the average size of hotspots in the *X* axis is larger than that in the *Y* axis and the hotspots exhibit an elliptical shape is mainly due to the linearly polarization state of the incident light. Moreover, the simulated Talbot distances of the distinct nanoaperture shapes make no discrepancy, which is in line with the theoretical calculation.

### Fill factor

**Figure 3:**
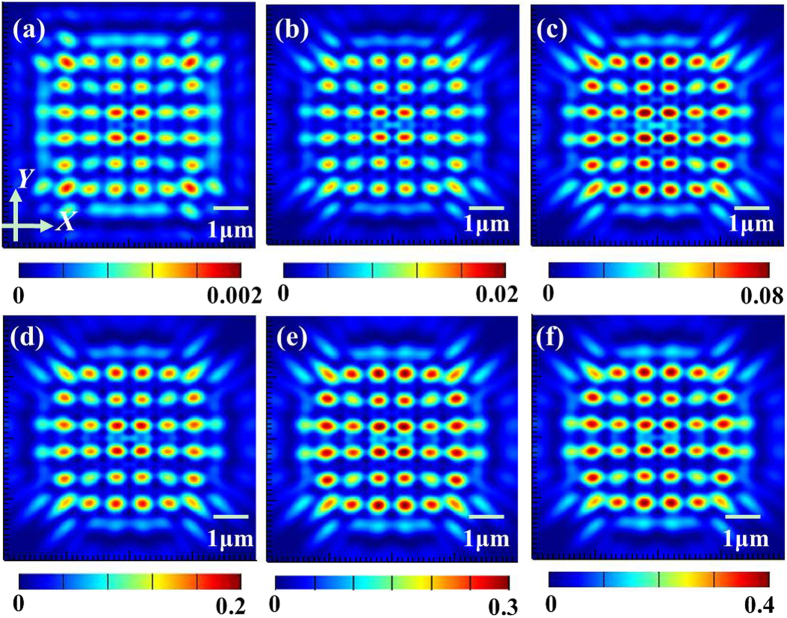
The simulated results of the output optical fields parallel to *X-Y* plane at the first Talbot planes. (**a**–**f**) Represent the fill factor of ~1.77%, 3.99%, 7.09%, 11.08%, 15.95% and 21.71%, respectively.

For the following discussions, the nanoholes were taken for the nanoaperture shape investigated. To study the impact of fill factor on the Talbot effect, we constructed the sample #2, composed of nanoholes in a square lattice penetrated in the gold film with the radii varying from 50 nm to 175 nm with a 10-nm interval, thus the corresponding fill factor varies from ~1.77% to 21.71%. Talbot *revivals* carrying abundant information of subwavelength hotspots can be captured at the first Talbot distance, as shown in Fig. 3, in which only six typical cases are given with the fill factor of ~1.77%, 3.99%, 7.09%, 11.08%, 15.95% and 21.71%, respectively. To get more information about the hotspots at the Talbot planes, the derived specific data of the hotspot size along the *X* axis and *Y* axis can be obtained in order to further analyze the subwavelength hotspots quantitatively.

**Figure 4:**
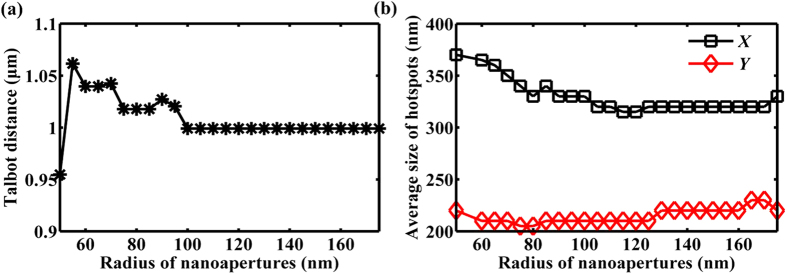
The derived (**a**) Talbot distance and (**b**) average size of hotspots for both axes at the first Talbot planes as the radius of nanoapertures increases. The black and red marked lines in (**b**) represent the *X* axis and *Y* axis, respectively.

From Fig. 3, we can see that the intensity of the light patterns at the first Talbot planes becomes gradually stable with the increase of fill factor. The same trend of the simulated Talbot distance and the average size of hotspots for both axes can also be observed, as shown in Fig. 4.

### Lattice distribution

**Figure 5:**
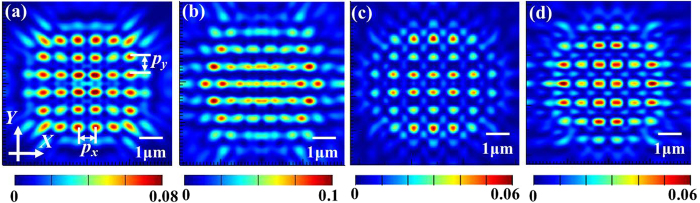
Light patterns at the first Talbot distance for (**a**) square lattice, *p*_*x*_ = *p*_*y*_ (**b**) triangular lattice (**c**) rectangular lattice, *p*_*y*_ = 1.5*p*_*x*_ and (**d**) rectangular lattice, *p*_*x*_ = 1.5*p*_*y*_.

In order to explore the influence of lattice distribution on the Talbot effect, we established a triangular-lattice sample and made the rectangular lattice with different horizontal period *p*_*x*_ and vertical period *p*_*y*_. Figure 5 gives the simulated light patterns at the first Talbot distance for different cases.

**Figure 6:**
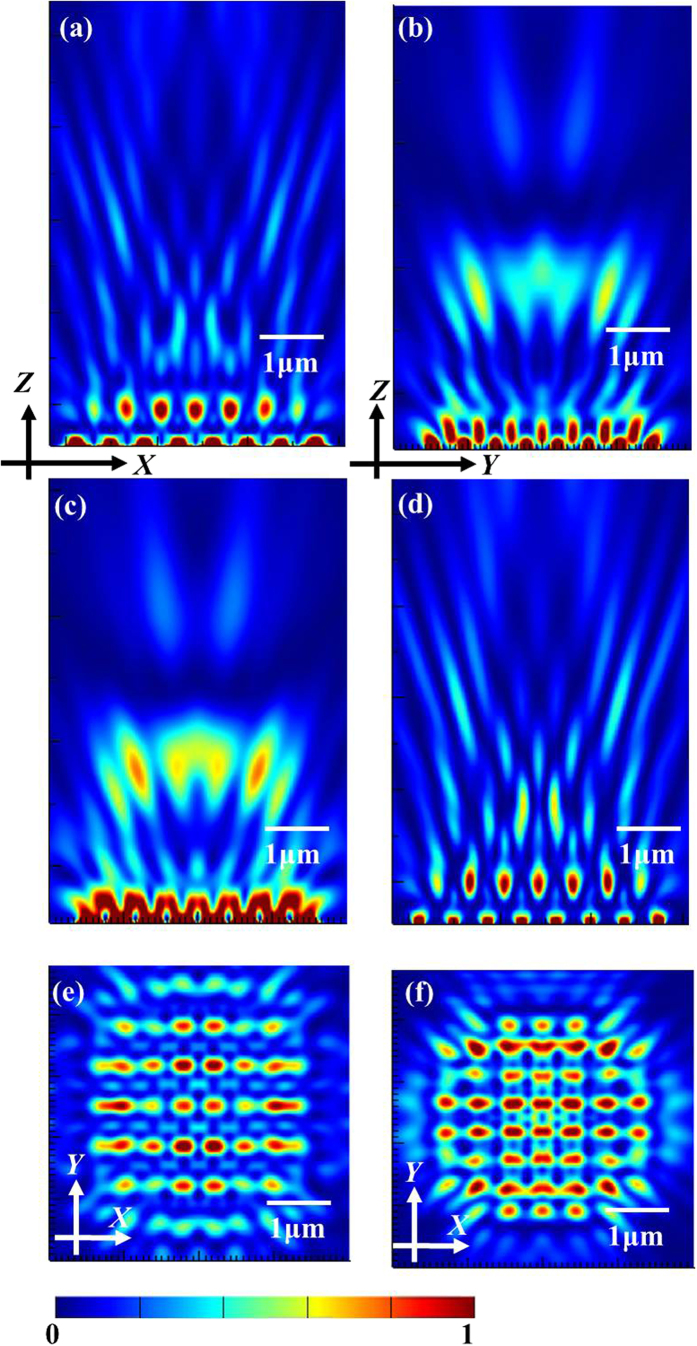
The output optical fields for: (**a** and **c**) the intensity contours for the cases *1 and *2 in the *X-Z* plane, respectively; (**b** and **d**) the intensity profiles for the cases *1 and *2 in the *Y-Z* plane, respectively; (**e** and **f**) the first Talbot light patterns for the cases *3 and *4, respectively.

We can clearly see that the light patterns of square lattice are the most apparent than the other lattice distributions. Although the triangular lattice distribution gives out a little higher light intensity, the electric-field distribution is not so uniform as the square lattice. From the equation (1), it is obvious that the effective wavelength of the incident light and the array period are the critical influencing parameters for the Talbot effect. However, when the array period doesn’t equal to each other along the *X* axis and *Y* axis, the theoretical Talbot distance cannot be directly derived by the above expression any longer. Then we conclude such an assumption that the theoretical Talbot distance is determined by the smaller period along the two axes. In order to verify our assumption, four cases with different axial array periods, denoted as *1, *2, *3 and *4, were modeled by the FDTD simulations, whose specific parameters can be seen in Table 2. From Fig. 6(a) and (b), we can see that the intensity distribution first replicating the structure pattern shows a sharp contrast for the two cross-sectional profiles in the *X-Z* plane and *Y-Z* plane for the case *1, bringing about the final Talbot contours determined by the more pronounced optical fields in the corresponding cross-sectional plane, and so it is for the case *2 (see Fig. 6(c) and (d)). Accordingly, the clear Talbot patterns at the derived Talbot planes for the cases *3 and *4 are shown in Fig. 6(e) and (f), respectively. Obviously, the light distributions of Talbot hotspots replicate the similar structure patterns. The theoretical and simulated Talbot distances for the considered four cases are listed in Table 2, which confirms our assumption. Therefore, the Talbot distances for rectangular arrays can be predicted by this way in the future device design.

### Array size

**Figure 7:**
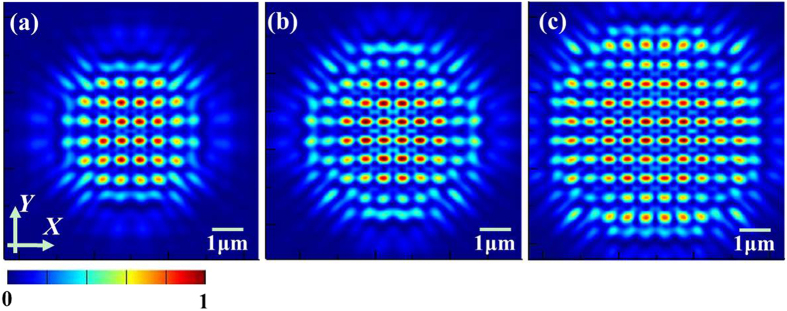
The simulated light patterns at the first Talbot planes for typical array sizes, (**a**) 8×8, (**b**) 10 × 10, (**c**) 12 × 12.

Concerning the practical application and efficiency, a gradually increasing array size was also investigated, meaning that the nanoaperture array was modulated from 8 × 8 to 18 × 18. We only give out the light patterns for the array size from 8 × 8 to 12 × 12 for clarity in Fig. 7, from which we can find out that the array size makes no clear difference for these distinct samples. It is noteworthy that the inner hotspots show the higher light intensity than the outer ones. This fringing effect makes the precise control over the Talbot effect generated by the finite-sized periodic arrays of metallic nanoapertures much more complicated. To quantitatively analyze the Talbot patterns of various device sizes, the average size of hotspots for the distinct samples is derived and listed in Table 3. It can be observed that when the array size becomes larger, the average size of hotspots for both axes almost remain unchanged in whole. Furthermore, the simulated Talbot distance as the array size increases is around 1.02 μm, which shows a good consistence with the theoretical result.

### Film thickness

**Figure 8:**
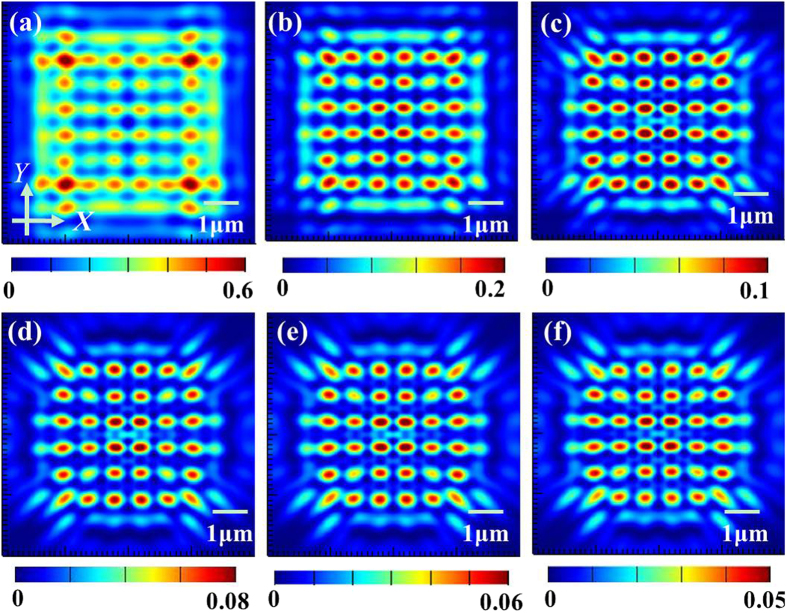
The simulated light patterns at the first Talbot planes for various metallic film thickness, (**a**) 50 nm, (**b**) 100 nm, (**c**) 150 nm, (**d**) 200 nm, (**e**) 250 nm, (**f**) 300 nm.

**Figure 9:**
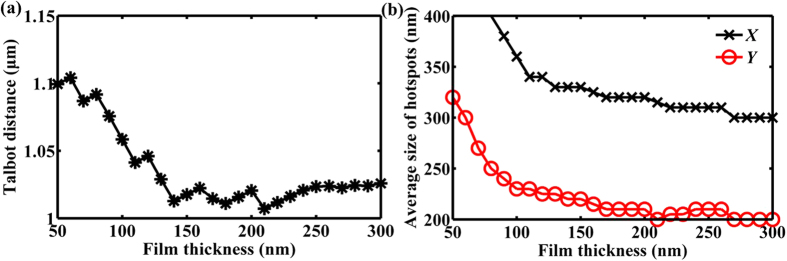
The derived (**a**) Talbot distance and (**b**) average size of hotspots for both axes at the first Talbot planes as the film thickness changes. The black and red marked lines in (**b**) represent the *X* axis and *Y* axis, respectively.

When the plane wave is incident from the back of a metallic film, the light is partially converted into surface plasmon polaritons (SPPs) at the exit surface of the film, contributing to the plasmon Talbot effect. Due to this mechanism, the plasmon Talbot effect is closely related to the thickness of the metallic film. We set up a series of samples with the different film thickness, from 50 nm to 300 nm at a 10-nm interval. We can obtain from the simulated light patterns of various film thickness in Fig. 8 that the light intensity for the 100-nm-thick sample is the largest among all the cases except the 50-nm-thick sample, revealing a relatively weak Talbot effect. Note that the transmission occurring for the 50-nm case can be due to the skin depth of Au. As the film thickness increases, the simulated Talbot distance gradually decreases and stays as a constant finally as seen from Fig. 9(a). Meanwhile, the average size of hotspots is reduced for both axes as the film thickness increases and gradually becomes stable as shown in Fig. 9(b).

### Material property

**Figure 10:**
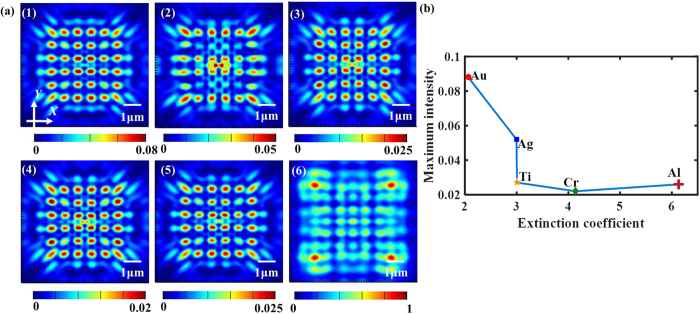
(**a**) The simulated light patterns at the first Talbot planes for various materials: (1) Au, (2) Ag, (3) Al, (4) Cr, (5) Ti, and (6) Si. (**b**) The relationship between the maximum intensity and extinction coefficient for various metallic materials.

Different films will act totally diversely when illuminated by the same incident light, revealing the different plasmonic behavior. Therefore, we surveyed the common metals by the simulations, including Au, Ag, Al, Cr and Ti. Moreover, in order to know the impact of plasmons produced by the metallic film, we also took Si film into account, a kind of material without plasmons for the operating wavelength. The light patterns at the first Talbot planes through the finite-sized nanoaperture arrays made of different materials are presented in Fig. 10(a), from which we can see that the output optical field parallel to the *X*-*Y* plane at the first Talbot planes created by the Au film is the most remarkable than the other materials, and it can be attributed to the different propagating loss in the materials. The energy intensity in the metal may decay exponentially and be closely related to the dielectric constant due to the fact that the electric density can be calculated as *E* = *E*_0_*e*^*−ikz*^ (*E*_*0*_, *k* and *z* is the initial electric density, wave vector of the plane wave and propagating length, respectively). According to the ref. 29, we can see that the energy loss can be related to the imaginary part of the dielectric constants for various materials, which are also correlated with the materials’ extinction coefficient. Furthermore, the influencing rules of the maximum intensity of the Talbot contour changing with the extinction coefficient can be acquired, as shown in Fig. 10(b), from which we can infer that the Talbot hotspots produced by Au film is the most pronounced because the extinction coefficient of Au is the smallest under the operating wavelength of 525 nm. However, Si film in the same case cannot achieve the relatively perfect self-imaging *revivals* for the original array as the metallic films do. Furthermore, the average size of hotspots and the simulated Talbot distance for the distinct materials are listed in Table 4. It can be figured out that only the simulated Talbot distance of Au film shows a highly consistence with the theoretical result, while the other kinds of metals come out with a slightly changed Talbot distance. Moreover, the average size of hotspots for both axes seems to make no difference with regard to the materials focused in our research.

### Polarization state of incident light

**Figure 11:**
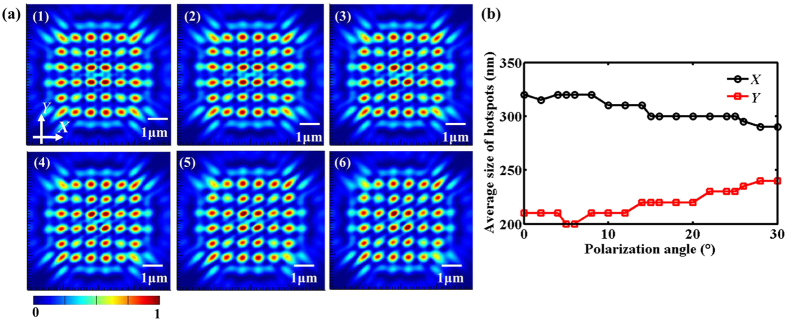
(**a**) The simulated light patterns for various polarization angles of the incident light, (1) 5°, (2) 10°, (3) 15°, (4) 20°, (5) 25°, (6) 30°. (**b**) Average size of hotspots for both axes at the first Talbot planes as the polarization angle changes. The black and red marked lines in (**b**) represent the *X* axis and *Y* axis, respectively.

Complex Talbot patterns can be observed when the finite-sized periodic arrays of metallic nanoapertures are normally illuminated by a coherent plane wave. When the device is illuminated by a linearly polarized beam, the change of polarization angle *θ* for the sample #5 as shown in Fig. 1(c) for the incident plane wave may play a role in the Talbot effect. Henceforth, we set the polarization angle *θ* from 0° to 30° with a 2° interval and the light contours at the first Talbot planes are given in Fig. 11(a). The simulated average size of hotspots at the first Talbot distance for both axes as the changed polarization angle was derived, as demonstrated in Fig. 11(b). Additionally, the simulated Talbot distance keeps a constant value of 1.02 μm, showing a good agreement with the theoretical result. It can be seen from Fig. 11(a) that a clear Talbot pattern can still be observed as the polarization angle *θ* is increased, causing the light patterns rotate along the *X* axis. Figure 11(b) tells us that the average size of hotspots in the *X* axis goes down while that in the *Y* axis goes up gradually as the polarization angle *θ* increases.

## Discussions

We comprehensively consider the possible factors, i.e. the nanoaperture shapes, fill factor, lattice distribution, array size, film thickness, material property and polarization state of the incident light that may influence the Talbot effect of the finite-sized periodic arrays of metallic nanoapertures by employing a full-wave electromagnetic simulation using the 3D FDTD method. As demonstrated in our previous research, the nanoaperture shapes seem to show no big difference to the Talbot patterns, which is also verified in this research. The simulated average size of hotspots at the first Talbot planes for distinct nanoaperture shapes exhibits a little diversity. Furthermore, it can be obviously seen that the light intensity is enhanced with the increase of fill factor, but shows slight influence on the average size of hotspots for both axes. When it comes for practical applications, the impact of device size becomes more and more important. It can be figured out that the Talbot contours reveal no difference as the array size is enlarged, while the inner hotspots possess the higher intensity due to the fringing effect and the results are in good accordance with the conclusion reported by other groups^30^,^31^. However, the experimental Talbot distance doesn’t remain unchanged as the results achieved in the simulations, attributing to the inevitable manufacturing and testing errors.

Another point worthy to note is that the square lattice seems to exert the most uniform transmitted optical field than the other kinds of lattice distribution, which has also been confirmed in the previous experiments. The interaction between the two adjacent nanoapertures cannot be ignored, and the surrounding SPP mode for a single nanoaperture differs for various lattice distributions. When it comes to a rectangular distribution, we found out that the Talbot distance can be estimated by taking the smaller axial period into consideration. By utilizing this feature, we can design a series of quasi-periodic structures for the actual use to fulfill the complex light patterns.

For the plasmon Talbot effect, the metallic film thickness and the film material deserve a certain concern due to its inherent physical mechanism. Various film thickness and metallic materials give out distinct output intensity but still explicit Talbot profiles. The average size of hotspots for both axes and the simulated Talbot distance stay constant as the metallic film thickness is increased, and the slight variation can be attributed to the material absorption and loss. Considering the skin depth of metallic materials, the thickness for Au film is suggested within the range of 50~100 nm for the illumination wavelength of 525 nm. Note that different metallic materials show different electromagnetic properties determined by their intrinsic dielectric constants, which may affect the light propagating property in the metallic materials.

**Figure 12:**
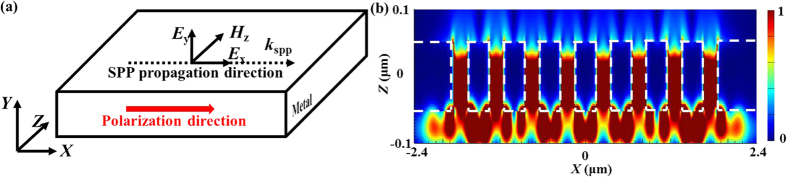
(**a**) Diagram of the excited SPP propagation along with the polarization direction of the incident light. (**b**) The normalized intensity distribution in the cross section along A-A in Fig. 2(a).

The last but not the least, we consider about how the polarization state of the incident light influences the Talbot effect of the finite-sized metallic arrays. Clear Talbot contours can be obtained at the first Talbot planes when the device is illuminated as the polarization angle of the linearly polarized plane wave is changed, causing the light patterns rotate according to the polarization direction. We found out that the excitation of SPPs at the interfaces of metal and dielectric generally depends on the local polarization direction of the incident light, which shows a big difference to the hotpots’ shape in the Talbot patterns. It is well known that SPPs along the metal/dielectric smooth interface can be only excited by transverse magnetic (TM) polarization. When the plane beam linearly polarized in the *X* axis propagates along the *Z* axis, the excited SPP propagation direction can be seen in Fig. 12(a) and the corresponding electric intensity distribution can be captured in Fig. 12(b) where the cross section was selected from *A-A* in Fig. 2(a). This explains the phenomenon that when the device is illuminated by the changing polarization angle of the linearly polarized plane wave, the corresponding light patterns rotate according to the polarization direction. One point should be paid much attention to is that the hotspots at the Talbot planes generated behind the nanoholes look like the elliptical shape for the reason that LSPR mode changes as the local polarization direction of the non-circular symmetrical electromagnetic field distribution due to the linear polarization incident light. Therefore, the corresponding intensity distribution is sensitive to the polarization direction, making the hotspots rotate along a certain axis and the derived average size is enlarged in the *Y* axis while that in the *X* axis is diminished simultaneously. In order to acquire a circular symmetrical hotspot in the Talbot plane, the radially and circularly polarized plane wave can be utilized for the further design. In addition, the polarization direction seems to show tiny influence on the simulated Talbot distance.

## Conclusions

To sum up, this paper focuses on a preliminary simulation investigation of the far-field optical transmission properties through the finite-sized 2D periodic arrays of metallic nanoapertures. The parameters that may influence the Talbot effect were studied in detail via the 3D FDTD numerical simulation method, including the shape of nanoapertures, fill factor, lattice distribution, array size, film thickness, material property, and polarization state of the incident light. The Talbot effect forming a large number of subwavelength light hotspots in the output medium was observed and the detailed hotspots’ size for both axes was deduced. The simulation results show that for the same device pattern, fill factor does not seem to exhibit a big difference for the Talbot effect, as well as the array size. Furthermore, the triangular lattice leads to a weaker Talbot effect than the square lattice, and the Talbot distance for rectangular distribution is suggested. Additionally, the nanoaperature shape seems to play a little role in the subwavelength hotspots. These summarized rules in the paper are in good agreement with our previous work. Moreover, the thickness of metallic Au film is recommended to be within the range of 50~100 nm for the illumination wavelength of 525 nm and shows a broadest Talbot pattern than the other metals discussed due to their various dielectric constants. It is also found out that the shape of subwavelength hotspots in the Talbot planes shows a polarization-dependent characteristic with the light source. In other words, the long axis of the elliptical hotspots is in accordance with the polarization direction of the linearly polarized light source. This study provides a valuable reference for the design of periodic arrays of metallic nanoapertures to exhibit the Talbot effect. Admittedly, a further study considering the other factors such as the angle of incident light and the ratio of period to wavelength can be deployed. Furthermore, the fabrication and characterization errors, as well as the efficiency optimization should be paid more attention to in the future research.

## Additional Information

**How to cite this article**: Li, W. *et al*. Investigation on the plasmon Talbot effect of finite-sized periodic arrays of metallic nanoapertures. *Sci. Rep.* **7**, 45573; doi: 10.1038/srep45573 (2017).

**Publisher's note:** Springer Nature remains neutral with regard to jurisdictional claims in published maps and institutional affiliations.

## Figures and Tables

**Table 1:** The simulated Talbot distance *τ* and average size of hotspots for various nanoaperture shapes.

		circle	cross	ellipse	ring	square	triangle
Average size of hotspots (nm)	*X*	320	320	310	320	310	320
	*Y*	210	220	230	210	230	230
*τ*_*sim*_ (μm)		1.02048	1.02048	1.02048	1.02048	1.02048	1.02048

**Table 2:** The theoretical and simulated Talbot distance *τ* for various cases.

Case	Array period along each axis (nm)		*τ* (μm)	
	*X*	*Y*	*τ*_*th*_	*τ*_*sim*_
*1	750	500	1.02	0.95
*2	500	750	1.02	1.00
*3	500	450	0.76	0.80
*4	500	650	1.02	0.98

**Table 3:** The simulated average size of hotspots for various array size.

		8×8	10×10	12×12	14×14	16×16	18×18
Average size of hotspots (nm)	*X*	320	330	320	320	330	310
	*Y*	210	210	210	220	210	210

**Table 4:** The simulated Talbot distance *τ* and average size of hotspots for various materials.

		Au	Ag	Al	Cr	Ti
Average size of hotspots (nm)	*X*	320	320	310	320	310
	*Y*	210	220	220	210	210
*τ*_*sim*_ (μm)		1.02048	0.954736	0.932819	0.954736	0.954736
